# The Action, Therapeutic Value and Use of the Carlsbad Sprudel Salt

**Published:** 1891-09

**Authors:** 


The Action, Therapeutic Value, and Use of the Carlsbad Sprudel Salt (Powder Form), and its Relation to the Carlsbad Thermal Water. By Dr. W. Jaworski, Demonstrator at the University of Krakow. Clinical experiment researches made at the University Clinic of Professor Korczynski, in Krakow, with a Dietary by the translator, A L. A. Toboldt, M. D., Assistant Demonstrator of Pharmacy, University of Pennsylvania; Editor *Journal of Balneology and Medical Clippings.* 8vo, pp. viii.—100. Philadelphia: P. Blakiston, Son & Company. 1891.

The importance of an understanding of the subjects treated of in this book is constantly on the increase. Physicians are more and more growing into the habit of prescribing mineral waters in the management of disease; hence, it is important that the therapeutic action of these waters shall be well understood. Carlsbad waters have occupied a deservedly high place in treatment of various maladies, and we advise those who are not already familiar with their therapeutic value to purchase this treatise.

